# Worse Theory of Mind in Only-Children Compared to Children With Siblings and Its Intervention

**DOI:** 10.3389/fpsyg.2021.754168

**Published:** 2021-11-25

**Authors:** Zhuo Zhang, Haoxue Yu, Muyun Long, Hui Li

**Affiliations:** ^1^School of Education, Central China Normal University, Wuhan, China; ^2^School of Education, University of Glasgow, Glasgow, United Kingdom

**Keywords:** theory of mind, first-born children, second-born children, siblings, only-children, cognitive verb training

## Abstract

The purpose of this study was to explore theory of mind (ToM) differences in children with different birth orders (only-children, first-born children, and second-born children), and further explore the effect of cognitive verb training for only-children’s ToM. Adopting the paradigm of false belief, Study 1 was conducted in which a sample of 120 children aged 3–6, including first-born children, second-born children (siblings aged 1–13 years), and only-children were tested. The results showed that (1) children aged 3–6 had significantly higher scores on first-order false-belief than second-order false-belief. (2) Controlling for age, the only-children scored significantly lower than the first-born children. In Study 2, 28 only-children aged 4–5 (13 in the experimental group and 15 in the control group) who initially failed in false-belief tasks were trained with the cognitive verb animations. Significant post-training improvements were observed for only-children who received training of animations embedded with cognitive verb. Those findings indicated that ToM of only-children was significantly worse than first-born children of two-child families, and linguistic training could facilitate ToM of only-children whose ToM were at a disadvantage.

## Introduction

China’s one-child policy was set in place in 1979 and was in force until about 2013. Since then, the country’s family planning guidelines have evolved, and the number of non-only-children families has gradually increased. According to statistics from the National Bureau of Statistics of China (NBS), there was a 1.62 million increase in second-born children in 2017 compared with 2016, and the second-born child birth rate accounted for 62.17% of the country’s total birth rate in 2019 (Li, 2019; Zhang, 2020).

The traditional family structure of only-child has changed with the new fertility policy in China. Many argued that children would benefit from having a sibling (Li et al., 2019a; Liu et al., 2019; Zhu et al., 2020). When a child experiences the birth of a younger brother or sister, the older child has a greater sense of responsibility and often comforts or takes care of younger siblings when their mother is away (Stewart, 1983; Paine, 2017; Liu et al., 2019). Older siblings are also more likely to take on the role of tutors, caregivers, and playmates, and consequently, their prosocial behaviors such as sharing, empathy (Li et al., 2019b), and cooperation (Hughes et al., 2018) are developed in the process of sibling interaction. Furthermore, being a younger sibling with older siblings also shows advantages in developing communication and interaction with others. The giving and cooperative behaviors of older children in sibling interaction positively predict the cooperative behaviors of younger children in sibling interaction (Dunn and Munn, 1986; Prime et al., 2017). Overall, researchers in China and in the West documented that children with siblings have better social skills (Hughes et al., 2018; Li et al., 2019b; Liu et al., 2019; Zhu et al., 2020). Older children learn to understand the emotions of younger siblings and how to take care of others through sibling interactions, and acquire social skills such as comforting and sharing (Liu et al., 2019). Younger children can also imitate the prosocial behaviors of older children in the sibling interaction, and thus show more prosocial behaviors (Dunn and Munn, 1986; Prime et al., 2017). In the current study, we examined whether having a sibling was also helpful for 3–6 years old children to develop theory of mind (ToM), and explored whether the development of children’s ToM could be improved through intervention training.

### Theory of Mind

Theory of mind is one aspect of children’s social cognitive development (Premack and Woodruff, 1978). ToM refers to the individual’s ability to speculate and comprehend others’ mental states of true and false belief, memory, imagination, and the like (McAlister and Peterson, 2007). ToM contributes to children’s self-cognition and interpretation of their own behaviors (Zeng et al., 2019), and an individual with ToM can causally explain others’ thoughts, feelings, and behaviors (McAlister and Peterson, 2013).

Over the past 20 years, children’s ToM based on cognitive belief has been the focus of many researchers (Onishi and Baillargeon, 2005; McAlister and Peterson, 2007; Paine, 2017), and there have been an abundance of evidence to suggest that children’s ToM gradually matured with age and social experience (Wellman et al., 2001; Zhang et al., 2004; Liu and Li, 2010). Researchers relied on implicit methods of non-verbal responses (e.g., anticipatory gaze) and found that individuals could make correct inferences about the cognitive beliefs of agents from infancy (Onishi and Baillargeon, 2005; Surian and Geraci, 2012). Kovács et al. (2010) pointed out that infants as young as 7 months could infer others’ beliefs and make correct inferences by visual fixation. After the age of 3, children began to infer others’ beliefs in explicit ways, including verbal and behavioral responses (Leslie, 1994; Wellman et al., 2001; Sang and Xu, 2006; Wiesmann et al., 2018).

As children grow older, the development of socio-cognitive skills through social experiences could contribute to develop a “mature ToM.” Children had deeper understanding of the cognitive state of others, from the first-order belief to the second-order belief (Perner and Wimmer, 1985; Zhang et al., 2004). The former refers to understanding beliefs about true events (e.g., A believes the ball is in the basket) and the latter one is an inference about other’s thought (e.g., A believes that B believes the ball is in the basket) (Liu and Li, 2010). Children’s second-order false belief usually develops later than the first-order belief by around age 6 (Sullivan et al., 1994) and matures at the age of 8 (Liu and Li, 2010).

In summary, children’s correct reasoning about an individual’s belief of facts and beliefs of others develops with age (Zhang et al., 2004). The present study examined children’s ToM based on the cognitive belief and adopted false belief tasks to assess children’s ToM. The first goal of this study was to explore first-order false belief and second-order false belief in Chinese children aged 3–6.

### Siblings and Theory of Mind

Sibling environment contributes to the development of children’s ToM (Dunn, 2006; McAlister and Peterson, 2013). Several studies have found that children in multi-child families have better ToM than only-children (Jenkins and Astington, 1996; McAlister and Peterson, 2013; Devine and Hughes, 2016). For example, McAlister and Peterson (2013) conducted a 1-year study with children aged 3–5 and found that children with two or more siblings had more advanced ToM than only-children. A child with a sibling has a more complex communication environment, and more opportunities to interact socially, but an only-child has his/her communication and interaction at home only with adults (Dunn, 2006). More sibling interactions provided opportunities for older and younger children to understand the mental state of each other, which promoted children’s ToM in multiple-child families (Paine et al., 2018).

Researchers have noted that children’s ToM is related to their birth order among siblings in the family (Perner et al., 1994; Ruffman et al., 1998; McAlister and Peterson, 2007). Some studies have suggested that having an older brother or sister can promote children’s understanding of false beliefs, while having a younger sibling or a twin does not have the same positive effect (Perner et al., 1994; Ruffman et al., 1998). However, some researchers argued that having a younger sibling or being a twin can contribute to the development of children’s ToM, and whether siblings can promote children’s ToM depends on siblings’ age and the age gap between siblings (Peterson, 2000; McAlister and Peterson, 2013; Paine et al., 2018). McAlister and Peterson (2007) found children with two or more siblings (aged 1–12 years) scored significantly higher than those with no child-aged siblings. Paine et al. (2018) also found that the existence of a younger sibling predicted the advantage in first-born children’s false belief performance when first-born children experienced the arrival of a sibling after 2 years of age. Peterson (2000) argued that the low language ability of children younger than 1 year old prohibits them from communicating, thus their older sibling’s ToM is not strengthened. Also, if the age gap is too large, the older children’s role is similar to that of adults, therefore having a much older sibling would not provide typical sibling-related benefits for their ToM (McAlister and Peterson, 2013). Previous studies have shown that between 1 and 13 years of age, siblings may have an impact on the development of children’s ToM because they can provide the possibility of frequent interaction (Peterson, 2000; Cassidy et al., 2005; McAlister and Peterson, 2006). In the current study, we also limited the ages of the participants’ siblings to be between 1 and 13 years old.

According to the different family types (i.e., two-child families and only-children families) and children’s birth orders, children were divided into first-born children, second-born children, and only-children in the study. The second goal of the study was to assess the differences in children’s ToM between different birth orders (first-born, second-born, and only-child).

### Cognitive Verbs and Theory of Mind

Other than the effects of siblings, children’s ToM is closely related to the mental state language (Ebert et al., 2017, 2020; Grazzani et al., 2018; Roby and Scott, 2018; Devine and Hughes, 2019). Mental state language refers to the language used to describe the cognitive state of others, mainly including cognitive verbs (e.g., think and want) and syntax (e.g., A thought that B went shopping) (Hale and Tager-Flusberg, 2003; San Juan and Astington, 2017).

A few studies have documented that children’s ToM was improved by exposure to the mental state language that involved cognitive verbs (e.g., want, like, and think) (Devine and Hughes, 2019; Ebert et al., 2020). For example, Devine and Hughes (2019) tracked children aged 3–4 and their parents across a 13-month period, and the results showed that children exposed to high levels of parental mental state talk outperformed their peers in false belief understanding. A longitudinal study conducted on 224 children aged 3 and 4 for 1 year, and pointed out that parental mental state language predicted children’s ToM (Ebert et al., 2020).

Sentences embedded with cognitive verbs, as the form of sentential complement, were a necessary prerequisite for children to acquire ToM (Hale and Tager-Flusberg, 2003). Researchers further carried out cognitive verb training to investigate the effect of cognitive verbs on children’s ToM (Tager-Flusberg, 1997; San Juan and Astington; 2017). Cognitive verb training refers to presenting a context embedded with cognitive verbs for children (Lohmann and Tomasello, 2003). San Juan and Astington (2017) conducted the cognitive verb training that included animated language contexts embedded with cognitive verbs for children aged 2–4, and found that cognitive verb training did not promote children’s performance in explicit false belief tasks based on verbal responses. This may be due to the age of participants. Children before the age of three had difficulty responding to false belief questions based on verbal responses. Studies have pointed out that 4 –5 years old is the key period in children’s explicit ToM (Sang and Xu, 2006; Liu et al., 2017). As such, based on the study of San Juan and Astington (2017), our study further adopted children aged 4–5 as participants, and the third goal of this study was to test whether cognitive verb training (i.e., linguistic context containing cognitive verbs “think”) could promote the ToM of children aged 4–5 that was at a disadvantage.

### Current Research Overview

The aims of current research were to explore the differences of first-order false belief, second-order false belief and total ToM score in children aged 3–6 with different birth orders (Study 1) and to further explore the effect of intervention with the cognitive verb “think” on ToM (Study 2). We expected that (1) first-order false belief will be better than the second-order false belief; (2) those children with a sibling in two-child families will have better ToM than only-children. (3) Cognitive verb training will help improve only-children’s ToM.

## Study 1

### Participants

The participants were 109 children aged 3–6 years in Wuhan, China. All children came from middle-class families, and did not have history of neurological or psychiatric illness. The data were collected over a month. As part of the selection process, we used children’s family information questionnaires filled by the parents to select participants. Additionally, first-born children or second-born children participated in the study according to two criteria: (a) siblings of children were between the ages of 1 and 13 years old and (b) there were no twins. Only-children were those who have no biological siblings. During the experiment, eleven children were excluded from the sample due to lack of attention or comprehension of the tasks, or unwillingness to finish the tasks. Finally, complete data were obtained from 109 children (60 boys and 49 girls). Children ranged in age from 3 years 3 months to 6 years 2 months (*M_*age*_* = 55.34 months, *SD* = 11.44). The sample was divided into three groups: first-born children (*n* = 36; *M*_*age*_ = 58.56 months), second-born children (*n* = 36; *M*_*age*_ = 52.86 months), and only-children (*n* = 37; *M*_*age*_ = 54.62 months). There were no significant age differences among conditions for either age group. In the first-born group, the siblings’ ages ranged from 12 to 50 months. In the second-born group, the siblings’ ages ranged from 47 to 147 months.

### Measurement

#### Children’s Family Information Questionnaire

The children’s family information questionnaire was used to select participants who met the requirements of this study. Parents provided information about each child’s birth date, gender, and birth order (first-born, second-born, or the only-child) and his/her sibling’s birth date and gender.

#### Unexpected Content Task

A modified version of the unexpected content task (Hogrefe et al., 1986) was used as one measure of ToM (Song and Volling, 2018; Wang et al., 2019). A cookie box and a pencil were used. The experimenter presented the child with a familiar cookie box, asked the child what it was, then opened the box and showed the child the pencil in the box. Then the experimenter asked the child four questions. Two were comprehension questions (i.e., “Do you remember what you believed was in the box before you opened it? What did you see in this box after you opened it?”). Children’s answers to comprehension questions were not scored. The comprehension questions were asked to ensure that children understood the story, and see if the child could correctly identify the cookie box and the pencil. The experimenter helped the child who did not comprehend the task by prompting his/her answers. If the child still could not answer the comprehension questions correctly after prompting them three times, he/she would be excluded. The third question was the first-order false belief question (i.e., “If there is a child who has never opened this box before, what would he think is inside?”). The fourth question was the second-order false belief question (i.e., “What would the child think of what you believe is in the box?”). Each wrong answer was scored 0, and each correct answer was scored 1. Thus, the total possible scores ranged from 0 to 2. The experimenter asked the children not to touch the box during the experiment (in case they could figure out what was inside).

#### Unexpected Location Task

A modified version of the “Sally-Anne” task (Wimmer and Perner, 1983; Song and Volling, 2018; Wang et al., 2019) was used. There were a piglet toy and a bunny toy, both of whom had Chinese names. The experimenter told the child that the piglet and bunny were playing with a ball. The bunny saw the piglet put the ball in the red box, and when the piglet went out, the bunny secretly took the ball out of the red box and hid it in the green box. Then the bunny left, and the piglet came back. The child was given five questions. Three of them were story comprehension questions (i.e., “Which box did the piglet put the ball in? Which box is the ball in now? Does the piglet know the bunny has moved the ball?”). The fourth was a first-order false belief question (i.e., “Which box will the piglet look in to find the ball?”). The fifth was the second-order false belief question (i.e., “Which box does the piglet think the bunny will look in to find the ball?”). The comprehension questions were asked to ensure that children understood the story. The scoring rule was the same as that in the unexpected content task.

### Procedure

The study was conducted in a quiet room in the kindergarten. In the unexpected location task, the experimenter provided drawings and told the story. In the unexpected content task, the experiment provided real objects and told the story. The child who interrupted to comment or could not answer these three comprehensive questions correctly was redirected or told the story again. If the child still could not understand the content of the story after it was told three times, he/she was excluded.

The study was approved by the Research Ethics Committee of the University. Parents provided written consent for their children to participate. Each child was tested separately in a quiet room in their school. After Study 1 finished, children received a small sticker to thank them for their participation.

### Results

A *post hoc* power analysis was examined using G*Power 3 (Faul et al., 2007), which revealed that the power (1-β) of Study 1 (including 109 participants) was 0.84 with α set at 0.05 and the number of groups and measurements set at 3. The correlation analysis of unexpected content task scores and the unexpected location task scores (*r* = 0.86, *p* < 0.01) provided evidence of the concurrent validity of each task as a measure of children’s understanding of false beliefs.

The independent variables of this study included first order ToM scores, second order ToM scores, and total ToM scores, which were calculated by first-order false belief scores plus second-order false belief scores. Table 1 shows descriptive information about the scores of first-order false belief and second-order false belief, total ToM scores for first-born children, second-born children, and only-children. A paired-sample *t*-test was used to compare the first-order and second-order scores, which both had a potential range of 0–2. We found that children’s first-order ToM scores (*M* = 1.05, *SD* = 0.74) were significantly higher than the second-order ToM scores (*M* = 0.63, *SD* = 0.68, *t* = 5.26, *p* < 0.001, Cohen’s *d* = 0.59). An independent sample *t*-test showed no significant gender difference in the first-order ToM (*t* = 1.51, *p* = 0.13), the second-order ToM (*t* = 1.42, *p* = 0.16) or the total ToM scores (*t* = 1.81, *p* = 0.08).

**TABLE 1:** Descriptive data about the scores of first-order false belief, second-order false belief and ToM for first-born children, second-born children, and only-children.

	First-born children (*n* = 36)	Second-born children (*n* = 36)	Only-children (*n* = 37)
	
	*M (SD)*	*M (SD)*	*M (SD)*
First-order false belief score	1.25 (0.73)	1.00 (0.79)	0.89 (0.66)
Second-order false belief score	0.83 (0.78)	0.64 (0.68)	0.43 (0.50)
Total ToM score	2.08 (1.32)	1.64 (1.20)	1.32 (0.78)

The results showed that there was no significant age difference in three different birth order groups (*F* = 2.40, *p* = 0.10, $\eta_{\text{p}}^{2}=0.03$). We used age as a control variable to analyze the ToM of children with different birth orders. The result of covariance analysis showed that there was a significant difference in total ToM scores (*F* = 3.59, *p* < 0.05, $\eta_{\text{p}}^{2}=0.06$) in terms of birth order. Compared to only-children, first-born children had significantly higher total ToM scores (*p* < 0.05). The scores of second-born children were not significantly different from those of only-children (*p* > 0.05) or first-born children (*p* > 0.05).

### Discussion

We focused our investigation on testing the differences of ToM in children aged 3–6 with different birth orders, and whether children with a sibling had a stronger ToM.

The first hypothesis that first-order false belief developed better than the second-order false belief of children aged 3–6 was supported. The finding was consistent with earlier studies (Wiesmann et al., 2018; Marta et al., 2019). Generally, most of the children in the sample could make correct inferences about true events, but their abilities of inferring others’ beliefs were still developing.

Our study also found that first-born children who had one younger sibling in two-child families had better ToM than only-children. Firstly, compared with only-children, the interaction between first-born children and their siblings provided more opportunities to understand others’ cognitive beliefs; secondly, it may be closely related to the attitude about child-rearing (Yagmurlu et al., 2005; Ameneh, 2015). Since ancient times, Chinese parents have strongly emphasized that older children should take care of younger siblings. The concept may have given the first-born children more responsibility for the upbringing, and more observation and speculation of the cognitive state and behavior of the second-born children in the interactions between siblings. To this extent, compared with only-children, first-born children gained experience with the skills that were needed to develop ToM. Future research can conduct more explorations in the relationship between the parental upbringing of siblings and children’s ToM (Pauker et al., 2016).

There were no significant differences in total ToM scores between second-born children and only-children, which was inconsistent with our hypothesis. Two possible reasons were discussed to explain the result: first, the age gap between siblings may affect ToM of second-born children. The content and quality of interactions and games between siblings with different age gaps are different (Peterson, 2000; Cassidy et al., 2005; McAlister and Peterson, 2006). The differences of sibling interaction may affect the development of ToM for second-born children. Future work should examine the effect of different age gaps between siblings on second-born children’s ToM. Alternatively, it was possible that only one brother or sister provided fewer learning opportunities for the second-born child. Previous studies have identified that the greater the number of brothers and sisters, the more opportunities for interaction between siblings (Jenkins and Astington, 1996; Devine and Hughes, 2016). Interacting with an older sibling may not be enough to help the second-born child fully understand the cognitive state of others (McAlister and Peterson, 2013). Moreover, the cognitive development of older children was more “mature” than that of younger children, so the cognitive beliefs of older children may be too advanced for younger children to understand. Therefore, there was insufficient opportunity for second-born children to understand the mental state of others.

The above results suggested that only-children’s ToM was significantly worse than that of first-born children. Four to five years old is the key period in children’s development of ToM (Liu et al., 2017; Wiesmann et al., 2018). Therefore, our second study aims to examine the effect of cognitive verb training for only-children aged 4–5 who failed to pass false belief tasks.

## Study 2

### Participants

The participants consisted of 32 only-children aged 4–5. A total of four children who were distracted or absent in the post-test was excluded, and 28 valid data (14 girls) were obtained. The participants were assigned to one of two conditions: the experimental group from Study 1 received the cognitive verb training (*n* = 15, *M*_*age*_ = 54.40 months, *SD* = 3.20), the children who were re-recruited of the control group did not receive training (*n* = 13, *M*_*age*_ = 54.92 months, *SD* = 4.97). None of the participants had been exposed to the experiment animation videos before the experiment.

### Procedure

The cognitive verb training took place in a quiet room in the kindergarten. A female experimenter and the participant sat on the side of the table to watch the animations. An iPad Air2 with a resolution of 2048 × 1536 was used to play the animations. The experiment was completed in 20 days. Children in the control group did not receive cognitive verb training and participated in class activities normally. Our study had three sessions, as follows:

1. *Pre-test assessment:* The experimenter assessed only-children’s language ability, and tested false-belief understanding of only-children by unexpected location tasks. When the children gave the incorrect response about belief questions of unexpected location tasks, the experimenter only recorded the answers and did not make a response to children’s answers. Only-children aged 4–5 who failed in at least one unexpected location task were eligible for training.
2. *Cognitive verb training:* The training was conducted once a week for 10–15 mins, lasted for 2 weeks. There were six animations (three true beliefs; three false beliefs) for each training session. The training animations were played in a fixed random order.
3. *Post-test assessment:* The experimenter tested children’s ToM through two unexpected location tasks.

When the post-test was finished, children received a small sticker for their participation.

### Materials and Methods

#### Language Measures

The Peabody Picture Vocabulary Test, Fourth Edition (PPVT-4) was used to assess the linguistic ability of only-children. The materials were compiled and revised by Dunn and Dunn (2007) and later translated and revised in Chinese by Guo et al. (2019). Cronbach’s α reliability coefficients of the Chinese version of the PPVT-4 was above 0.90, and the test-retest reliability across 2 weeks was between 0.88 and 0.95, showing good reliability and validity (Guo et al., 2019).

Peabody Picture Vocabulary Test, Fourth Edition is a standardized test of receptive language ability (Milligan et al., 2007), and it is a booklet composed of words and pictures. During the test, the experimenter said a word and asked the child to point out the most suitable picture on the page of the assessment booklet or say the number of the picture. Each wrong answer was scored 0, and each correct answer was scored 1. No time limit was given for the test and the child was allowed to guess the answer.

#### Training Task

**FIGURE 1:**
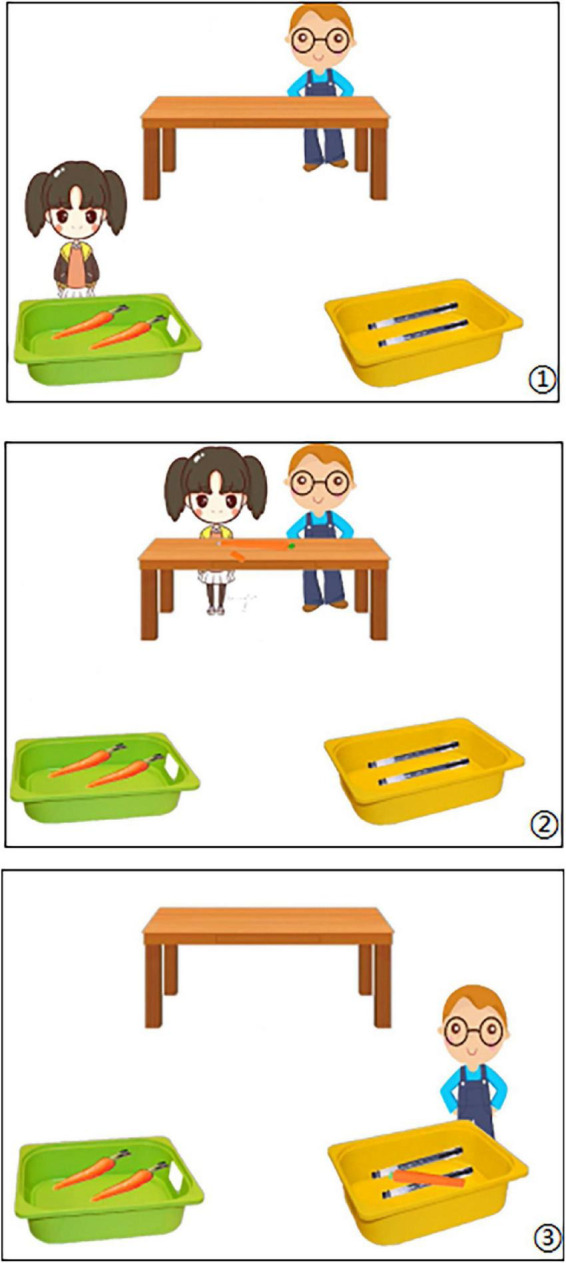
Example for cognitive verb training.

Cognitive verb training materials were adapted from the study of San Juan and Astington (2017). The training materials consisted of 12 animations, which comprised similar actions to describe scenarios of Appearance Reality (Figure 1). Each animation initially contained two types of objects that were placed in separate boxes (e.g., one box had carrots while the other had pens). Then, a deceptive appearance target object appeared in the table during the animation. The target object had the same appearance as the objects in one box, but its function was the same as the objects in the other box. There were two characters, A1 and A2 in six animations, the character A2 witnessed the function of the target object and therefore had a true belief about its identity. In the other six animations, the A2 did not witness the function of the target object and therefore had a false belief about its identity.

In each animation, character A1 introduced two types of objects from boxes (e.g., “Look, these are pens.”). Then the target object appeared on the table. A1 presented the function during each description of the target object (e.g., “Look, this is a carrot-like pen.”), and A1 exited the scene.

In the true-belief animations, A2 walked from the side of the scene to the table and saw the target object. Children then heard the statement that described the intentions of A2 [e.g., “This is (A2), (A2) sees the object on the table. (A2) wants to put it in one of the boxes.”]. Next, A2 placed the target object in one of the boxes. Children would hear descriptions containing the cognitive verb “think” to describe true distributive behavior of A2 [e.g., “(A2) thinks it is a pen, and it is indeed a pen.”].

In the false-belief animation, A2 walked from outside the scene to the table and saw the target object. The children also heard the statement that described the intentions of A2. Next, A2 placed the target object in one of the boxes. Children would hear descriptions containing the cognitive verb “think” to describe false distributive behavior of A2 [e.g., “(A2) thinks it is a carrot, but actually it is a pen.”].

After each animation, the children were asked behavioral questions (e.g., “Did A2 place it in the right box?”). All children needed to provide feedback and reply [e.g., “No, (A2) put it in the wrong box. It should be placed in the other box because it is a pen” or “Yes, (A2) put it in the right box because it is a pen”). If children did not provide feedback and reply, the experimenter repeated the question and waited patiently for the child to answer.

#### Theory of Mind Task

The unexpected location tasks, which had the same structure as in Study 1, were used in the form of animations to evaluate the ToM of children. The pre-test included two unexpected location tasks, and children who failed at least one of the two unexpected location tasks were eligible for the study. The post-test also included two unexpected location tasks, which had different story protagonists, but with a similar structure to the pre-test. The experimenter only asked children about story comprehension questions and the first-order false belief questions. The scoring rule was the same as for Study 1. The score ranged from 0 to 2 in pre-test and post-test. For evaluating the improvement of children’s ToM, the total ToM score was calculated by subtracting the pre-test correct scores from the post-test correct scores.

### Results

The *post hoc* power analysis was examined using G*Power 3 (Faul et al., 2007), which revealed that the power (1-β) of Study 2 (including 28 participants) was 0.83 with α set at 0.05 and the number of groups and measurements set at 2.

Table 2 displays the descriptive data of experimental group and control group at pre-test, post-test, and total ToM score. At pre-test, we randomly assigned participants to the experimental group and the control group, an independent *t*-test showed there were no differences in age (experimental: *M* = 54.27, *SD* = 0.76 and control: *M* = 54.46, *SD* = 1.16). For the scores of linguistic ability, the independent *t*-test showed that there were no differences between the experimental group (*M* = 101.00, *SD* = 5.90) and the control group (*M* = 108.85, *SD* = 5.61, *t* = 0.96, *p* = 0.35).

**TABLE 2:** Descriptive data of experimental group and control group at pre-test, post-test, and total ToM score.

	Experimental group (*n* = 15)		Control group (*n* = 13)	
	*M*	*SD*	*M*	*SD*
Pre-test score	0.27	0.46	0.46	0.52
Post-test score	1.00	1.00	0.23	0.60
Total ToM score	0.73	0.96	−0.23	0.60

The data were analyzed with 2 (group: experimental or control) × 2 (time: pre-test or post-test) design. A repeated measures analysis showed that there was a significant interaction between group and time factors, *F*(1,12) = 16.56, *p* < 0.01, $\eta_{\text{p}}^{2}=0.58$. Further simple effect analysis showed that only-children in the experimental group scored higher on the post-test false belief score than on the pre-test score, *F*(1,12) = 8.96, *p* = 0.01, $\eta_{\text{p}}^{2}=0.43$, and on the post-test score, only-children in the experimental group scored significantly higher than in the control group, *F*(1,12) = 11.52, *p* < 0.01, $\eta_{\text{p}}^{2}=0.49$.

### Discussion

Study 2 focused on examining the effect of intervention training with animations embedded with the cognitive verb “think” on children’s ToM based on cognitive belief. As expected in Hypothesis 3, children who participated in cognitive verb training performed better on ToM tasks of post-task than pre-test and compared to the control group. The results showed that observing and listening to others’ conversations containing cognitive verbs was beneficial to children’s ToM. In fact, the structure of training tasks was different from the post-test ToM tasks (i.e., the unexpected location scenarios vs. the Appearance Reality scenarios). Therefore, the improvement in the post-test ToM tasks indicated that children could transfer knowledge involved the cognition of others’ mental state to distinct contexts of reasoning in ToM. However, our findings are at odds with a previous study. San Juan and Astington (2017) found that cognitive verb training had no significant effect on the performance of explicit oral tasks when children aged 2–4 reasoned about others’ epistemic states. Except for false belief tasks, the misinformation tests that deliberately misinformed the cognitive beliefs of others were used to test children’s ToM in the study of San Juan and Astington (2017). According to cognitive resources theory, the more complex the processing of stimuli, the more cognitive resources are occupied (Hockey, 1997). It may incur more processing demands to judge others’ beliefs involved in misleading representations, thus significant effects of training on explicit task performance may have not been observed. Secondly, as opposed to the younger age group in the study of San Juan and Astington (2017), the age of our participants was 4–5 years old. Four to five years old was a key period for the development of children’s ToM (Liu et al., 2017; Wiesmann et al., 2018), thus the training intervention of our study may be more effective and observable.

## General Discussion

Two studies were carried out to explore the differences in the ToM based on cognitive belief of children with different birth orders and the effects of cognitive language intervention. In Study 1, the unexpected content task and unexpected location task were used to evaluate ToM of children aged 3–6 years with different birth orders. The results showed that scores of first-order false beliefs were significantly higher than scores of second-order false beliefs, and only-children performed worse in ToM tasks than first-born children in two-child families. In Study 2, we further included cognitive verb animations as training tasks, with the aim of examining the effect of training on only-children who were disadvantaged in ToM. The pre- and post-test ToM tasks that probed cognitive belief aspect of ToM were used to examine the effect of animations embedded with cognitive verbs on the ToM of only-children aged 4–5 who failed in the false belief tasks. The results showed that cognitive verb training could improve the ToM based on cognitive belief of only-children.

### Birth Order and Theory of Mind

Children’s understanding of false beliefs seemed typical for this age group. On the whole, children’s scores for first-order false beliefs were significantly higher than their scores for second-order false beliefs. In terms of children’s understanding of second-order false belief, only 11% of children answered the second-level false belief questions correctly. This result is consistent with a Chinese study where nearly 80% of children aged 3–6 could not fully understand second-order false beliefs (Zhang et al., 2004). In the current study, the first-order false beliefs and the second-order false beliefs of children aged 3–6 did not develop at the same time. Compared to speculating on others’ perceptions of the true event, the cognitive inferences of second-order false beliefs involved two propositions, that is, a character’s knowledge about another character’s beliefs or intentions. Children’s ability to recursively think about others’ cognitive states was more difficult (Zhang et al., 2004).

This study also found that first-born children in two-child families had better total ToM score than only-children. Interaction and communication between siblings, such as teasing, commanding, consoling, conflict, and pretend play provided children with the cognitive state of siblings (Youngblade and Dunn, 1995; Slaughter, 2015), and children also had the opportunity to eavesdrop on or joined in conversations between parents and siblings (Dunn and Shatz, 1989), which enhanced the skills needed to develop ToM. Only-children would not have these experiences and their development of ToM could be delayed as a result.

In ancient China, Mencius said that “elder brothers are like fathers,” an idea that has continued to influence family life in contemporary China. In one interview study regarding Chinese parents’ concept of education in two-child families, a common sentiment was that “elder brothers or sisters should take care of younger ones” (Li et al., 2019a). In order to better take care of their younger siblings, the first-born children may need to observe the behaviors and infer the thoughts of second-born children, which also benefits the development of their ToM.

It was expected that second-born children’s ToM would be better than that of only-children, which it was not. This may have been caused by the age gap and number of older siblings. On the one hand, different age gaps between siblings may lead to differences in sibling interactions in the content and quality (Cassidy et al., 2005; McAlister and Peterson, 2006). On the other hand, there was only one older sibling for each second-born child, and sibling interaction and communication may be insufficient. As such, the ToM of second-born children failed to benefit from the interaction with first-born children. With the promulgation of the three-child policy in China, many families may soon welcome their third child. The transformation of the fertility policy was conducive to the research of ToM for children in three-child families. Future research can examine the differences of the development in ToM on children with two or more siblings.

### Training and Theory of Mind

The results showed that cognitive verb training improved the ToM ability of only-children who had failed in false-belief tasks. In the training tasks, true-belief situations and false-belief situations embedded with cognitive verbs were beneficial for children to recognize that individuals did not always have correct beliefs, and false beliefs could lead to incorrect assignment behaviors. San Juan and Astington (2012) pointed out that repeated exposure to the connection between verbs and context could help children abstract and clarify familiar patterns of reasoning (e.g., relations between an agent, an object and a subsequent series of actions). Sentences containing cognitive verbs likely helped children perform implicit processing of patterns by emphasizing the relationship between characters and their beliefs, and they may have further promoted the explicit verbal reasoning of individuals in other ToM tasks (San Juan and Astington, 2012).

The results of the intervention study not only provide educators and parents strategies to improve children’s ToM, but also have important value for populations with ToM impairments. In previous clinical studies, patients with traumatic brain injury (TBI) and schizophrenia had selective deficits in ToM, that is, TBI (Geraci et al., 2010) and schizophrenia (Geraci and Cantagallo, 2011) may result in an acquired impairment in representing and reasoning about mental states. For example, Bibby and McDonald (2005) evaluated performance in ToM tasks of TBI patients, and found that the clinical group performed worse on ToM tasks than the healthy group. Future studies can explore whether cognitive verb training can be used as means of rehabilitative treatments on improving ToM for patients with TBI.

## Limitations and Prospects

This study had some limitations that should be addressed. First, in Study 1, there was no task to assess language skills. Language ability was previously found to be related to children’s ToM (Astington and Baird, 2005; Milligan et al., 2007). Future studies could explore ToM of children in different birth orders based on controlling children’s language ability. Secondly, further work needs more experimental paradigms that measure the development of children’s ToM. We had only adopted the classic false belief experimental paradigm as a research tool to test children’s ToM. A variety of tasks are needed to focus on different aspects of ToM, including children’s understanding of others’ emotions and wishes (Fang et al., 2009). For example, researchers can use the “Belief-Emotion task” to test children’s perception of others’ emotions, or the “Diverse Desires task” that tests children’s understanding of others’ wishes (Wellman and Liu, 2010).

## Conclusion

This study contributes to understanding ToM of children with different birth orders under China’s two-child policy. On the one hand, the first-order false beliefs were better than second-order false beliefs of ToM in children aged 3–6, and only-children’s ToM based on cognitive belief was worse than that of the first-born children in two-child families. The findings provide empirical support for parents and educators to understand the development of ToM in children with different birth orders. On the other hand, we used cognitive verbs training that led to marked improvements in ToM of only-children. The positive impact of cognitive verb intervention provides an effective way to improve ToM of children, and older individuals with conditions associated with selective deficits in ToM.

## Data Availability Statement

The raw data supporting the conclusions of this article will be made available by the authors, without undue reservation.

## Ethics Statement

The studies involving human participants were reviewed and approved by the Human Research Ethics Committee, School of Psychology, Central China Normal University. Written informed consent to participate in this study was provided by the participants’ parents.

## Author Contributions

ZZ and HL designed and conducted the research and participated in the data analysis. ZZ and ML wrote the manuscript. HL and HY reviewed and revised the manuscript. All authors agreed to publish the final version of the manuscript.

## Conflict of Interest

The authors declare that the research was conducted in the absence of any commercial or financial relationships that could be construed as a potential conflict of interest.

## Publisher’s Note

All claims expressed in this article are solely those of the authors and do not necessarily represent those of their affiliated organizations, or those of the publisher, the editors and the reviewers. Any product that may be evaluated in this article, or claim that may be made by its manufacturer, is not guaranteed or endorsed by the publisher.
